# An endophytic *Schizophyllum commune* Fr. exhibits in-vitro and in-vivo antidiabetic activity in streptozotocin induced diabetic rats

**DOI:** 10.1186/s13568-021-01219-3

**Published:** 2021-04-21

**Authors:** Avinash Sharma, Rajvir Kaur, Jasleen Kaur, Saweta Garg, Rajbir Bhatti, Amarjeet Kaur

**Affiliations:** 1 Department of Microbiology, Guru Nanak Dev University, Amritsar, Punjab 143005 India; 2 Department of Pharmaceutical Sciences, Guru Nanak Dev University, Amritsar, Punjab 143005 India

**Keywords:** *Schizophyllum commune*, Basidiomycetes, α-Glucosidase inhibition, Antidiabetic

## Abstract

The present study aimed at isolation of endophytic basidiomycetous fungi and evaluation of their in-vitro and in-vivo antidiabetic potential. Preliminary screening for in-vitro activity was carried out using α-glucosidase inhibition assay. An endophytic isolate Sch1 (isolated from *Aloe vera)*, identified to be *Schizophyllum commune* Fr. on molecular basis, exhibiting more than 90% α-glucosidase inhibitiory activity was selected for further studies. Detailed in-vivo investigations for antidiabetic potential of ethyl acetate extract of *S. commune* (Sch1), at two different doses, were carried out in streptozotocin induced diabetic Wistar rats. Treatment of diabetic rats with *S. commune* extract caused significant decrease in blood glucose level and increase in body weight after 14 days experimental period. It significantly restored renal parameters including creatinine, blood urea nitrogen, fractional excretion of sodium, and potassium level in diabetic rats. Improvement in lipid profile and level of antioxidant parameters viz*.* reduced glutathione, thiobarbituric acid reactive species, and superoxide anion generation was also observed after treatment. Liver enzymes (serum glutamic pyruvic transaminase, serum glutamic-oxaloacetic transaminases, and alkaline phosphatase) homeostasis was found to be markedly improved in diabetic rats administered with *S. commune* extract. The effects were more pronounced at higher concentration and comparable to acarbose which was used as positive control. Phytochemical analysis revealed the presence of phenolics and terpenoids in the ethyl acetate extract. This is the first report highlighting the therapeutic potential of an endophytic *S. commune* in the management of diabetes.

## Introduction

Endophytes are microorganisms that reside within the living plant tissues for a variable period of their life without causing any symptomatic infection (Petrini 1991). Recently they have become the focus of attraction as a potent group of microorganisms able to synthesize diverse group of bioactive compounds. Various compounds derived from the endophytic fungi are known to exhibit a wide range of bioactivities like anticancer, antiviral, antidiabetic, antioxidant, immunomodulatory, antiparasitic, and antimicrobial (Strobel and Daisy 2003; Kaul et al. 2012; Kaur et al. 2020). A survey of literature revealed that majority of the bioactive compounds have been isolated from endophytic fungi belonging to the phylum Ascomycota. Endophytic basidiomycetes, despite their prodigious potential to produce bioactive molecules with application in various fields ranging from medicinal, agricultural, and industrial (Waller et al. 2005; Puri et al. 2006; Katoch et al. 2017), have not received much attention due to sampling biases. There are several reasons which restrict the detection of endophytic basidiomycetes with conventional culture-based method viz*.* slow growth and lack of knowledge about their morphological characters (Martin et al. 2015). Keeping in view their unexplored potential, we have attempted to isolate basidiomyceteous fungi from different plants and screened them for their bioactive potential. This study deals with the antidiabetic potential of endophytic basidiomycetes.

Diabetes mellitus, a group of metabolic disorders associated with elevated blood glucose level due to lack of insulin secretion, insulin resistance, or both, has emerged as a major health problem. It is statistically estimated that about 693 million people in the world are going to suffer from diabetes mellitus till 2045 (Cho et al. 2018). Diabetes mellitus results in severe complications such as neuropathy, nephropathy, retinopathy, and cardiovascular diseases (Clements and Bell 1985). Postprandial hyperglycemia is mainly responsible for the majority of the diabetes related complications (Gavin 2001; Ceriello 2005). Commercially available drugs used for the treatment of diabetes suffer from certain limitations and have side effects (Chaudhury et al. 2017), necessitating the need for novel and more effective antidiabetic agents from natural sources. One of the therapeutic approaches approved for the management of diabetes mellitus is the use of α-glucosidase inhibitors. By virtue of their ability to modulate the activity of carbohydrate hydrolysing enzymes, α-glucosidase inhibitors can help to control post-prandial hyperglycemia. In the present work, endophytic basidiomycetes were isolated and preliminary screening for their antidiabetic potential was conducted using in-vitro α-glucosidase inhibitory assay. After screening an endophytic culture, identified to be *Schizophyllum commune* Fr., possessing high in-vitro α-glucosidase inhibitory activity was selected and evaluated for its in-vivo antidiabetic effect in streptozotocin (STZ) induced diabetic rats.

## Materials and methods

### Isolation of endophytic fungi

Isolation of endophytic fungi was carried out from different healthy asymptomatic plants viz*. Aegle marmelos* (L.) Corr.*, Cordia myxa* L.*, Phyllanthus emblica* L.*, Lawsonia inermis* L.*, Ficus virens* Ait.*, Ocimum tenuiflorum* L.*, and Aloe vera* (L.) Burm.f.. Plant parts were thoroughly washed with tap water to remove dirt and solid particles. Surface sterilization was performed with 70% ethanol for 1–2 min, followed by 4% sodium hypochlorite exposure for 3–4 min, and subsequently washed with sterile distilled water. To ensure surface sterilization, the water obtained after last wash was plated on potato dextrose agar (PDA) (Himedia, Mumbai, India) plates. Surface sterilized plant parts were cut into small pieces (3–5 mm size) and placed on isolation medium containing malt extract (15 g/l), benomyl (50 mg/l), chloramphenicol (100 mg/l), streptomycin sulphate (100 mg/l), and agar (20 g/l) (Oses et al. 2008). The plates were incubated at 30 ℃ for 5–10 days and the emerging fungal mycelia were purified and preserved on PDA plates.

### Production

Purified endophytic fungal cultures were grown on PDA plates, and one mycelial agar plug (8 mm) from the periphery of the growing fungal cultures was inoculated in Erlenmeyer flasks (250 ml) containing 50 ml malt extract broth (malt extract 2%, dextrose 2%, peptone 0.1%). The flasks were incubated at 30 ℃ for 10 days in a rotary shaker at 180 rpm. After incubation, 50 ml ethyl acetate was added to each flask and extraction of metabolites was carried out at 40 ℃ for 1.5 h twice at 120 rpm. Thereafter, the upper organic phase was collected and concentrated to dryness by using rotary evaporator (BUCHI). The concentrated sample was then re-suspended in phosphate buffer saline (pH 7.4) for further use.

### α-Glucosidase inhibitory (AGI) assay

AGI assay was conducted using *p*-nitrophenyl-alpha-d-glucopyranoside (p-NPG) (Himedia, Mumbai, India) as substrate (Singh and Kaur 2016). In a 96 well plate, reaction mixture containing 50 μl phosphate buffer (50 mM, pH 6.8), 10 μl α-glucosidase (1 U/ml) (Himedia, Mumbai, India), and 20 μl extract was preincubated at 37 °C for five min. 20 μl p-NPG (2 mM) was added as substrate and incubated further at 37 °C for 30 min. The reaction was terminated by adding 50 μl Na_2_CO_3  _(0.1M). The end product of the reaction, *p*-nitrophenol formed after hydrolysis of p-NPG by α-glucosidase, was quantified by measuring the absorbance at 405 nm. Each experiment was performed in triplicates. Acarbose was used as a positive control. The results are expressed as percentage inhibition, which was calculated using the formula:

$$\% {\text{ inhibition}} = \frac{{{\text{Ac}} - {\text{As}}}}{{{\text{Ac}}}} \times 100$$

where A_c_ was the absorbance of control, and A_s_ was the absorbance of the sample.

### Identification of fungal culture

The culture was identified on molecular basis by amplification of ITS1-5.8S-ITS2rDNA region by National Centre for Microbial Resource (NCMR) Pune, India. Phylogenetic analysis was performed using MEGA-X software. Multiple sequence alignment of NCBI downloaded sequences was performed using clustalW software. Phylogenetic tree was constructed using “neighbor-joining” statistical method with bootstrap method for the test of phylogeny, considering 1000 number of bootstrap replications and Maximum Composite Likelihood as a substitution model.

The morphology of the selected isolate was observed under light microscope (Olympus). To determine the number of nuclei in per compartment of hyphae the fungal mycelium stained with 4′,6-diamidino-2-phenylindole (DAPI) (10 µg/ml) and observed under fluorescent microscope (Nikon ECLIPSE Ts2).

### Phytochemical tests

The fungal extract was subjected to various tests to determine the presence of different phytochemicals. 20 µl fungal extract was spotted on TLC silica gel and sprayed with different visualization reagents for the detection of alkaloids, phenolics, and amines. Dragendorff reagent was used to detect the presence of alkaloids. Detection of phenolics was carried out by spraying with Fast blue B (3,3-dimethoxybiphenyl-4, 4-bis(diazonium)-dichloride) and ferric chloride. Amines were detected using ninhydrin reagent. Terpenoids were determined by dissolving fungal extract in chloroform followed by addition of concentrated sulphuric acid (Waksmundzka et al. 2008; Batool et al. 2019).

## In-vivo studies

### Animals

Wistar albino rats aged between 8 and 10 weeks (170–200 g) were purchased from National Institute of Pharmaceutical Education and Research (NIPER), Mohali. Animal experiment protocol (226/CPCSEA 2017/03) was duly approved by Institutional Animal Ethics Committee (IAEC) of Guru Nanak Dev University, Amritsar, Punjab (India). All the experiments were conducted in accordance with the guidelines established by Committee for the Purpose of Control and Supervision of Experiments on Animals (CPCSEA), Ministry of Environment, Forests and Climate Change, Government of India. Wistar albino rats were housed in polypropylene cages under standard laboratory conditions of 23–25 $^\circ{\rm C}$ temperature with 12 h light–dark cycle and fed with standard pellet diet and water in the Animal House of Guru Nanak Dev University, Amritsar.

### Experimental design

A total of 25 Wistar albino rats were randomly divided into five groups with five rats each: normal healthy control group (NC), diabetic rats administered with STZ (Sigma-Aldrich, St. Louis, USA) (DC), diabetic rats treated with 60 mg/kg body weight of reference compound acarbose (SRL Pvt. Limited, India) (PC), diabetic rats treated with 50 mg/kg body weight (SC50) and 100 mg/kg body weight (SC100) of *S. commune* extract.

### Induction of experimental diabetes

After 15 days acclimatization of rats, a freshly prepared dose of STZ (45 mg/kg body weight) in 0.02 M citrate buffer (pH 4.5) was injected intraperitoneally to induce diabetes. To prevent the lethal effect of STZ induced hypoglycaemia, rats were administrated with 5% dextrose solution after STZ injection. On 4th day blood was drawn from tail tip, rats with blood glucose level higher than 400 mg/dl were considered diabetic and used for further study.

### Biochemical evaluation

From 4th day onwards oral administration of *S. commune* extract (SC50 and SC100) and standard drug acarbose was done at a fixed time (between 10:00 and 11:00 AM) till the 14th day. Blood sample for the determination of glucose level at 0, 4th, and 7th day was withdrawn from tail tip before the administration of *S. commune* extract, and blood glucose level was estimated by using electronic glucometer. At the end of experimental period, blood was drawn by retro orbital puncture from anaesthetized rats. Body weight of experimental animals was also recorded on 0, 4th, 7th, and 14th day. After 14 days experimental phase, rats were anaesthetized by intraperitoneal injection of ketamine (50 mg/kg). Blood samples were withdrawn by retro orbital puncture under anaesthesia before the rats were sacrificed by cervical dislocation. Serum samples were collected by centrifugation of blood and were used to estimate the level of creatinine, blood urea nitrogen (BUN), fractional excretion of sodium (FeNa), potassium, serum glutamic pyruvic transaminase (SGPT), serum glutamic-oxaloacetic transaminase (SGOT) and alkaline phosphatase (ALP). The quantification of lipid profile was also done in serum. Kidney and liver from all groups of rats were removed and washed with 1.17% potassium chloride (KCl) solution. A small part of both tissues was used to estimate SAG and the remaining section was minced and homogenized (10% w/v) in 1.17% KCl solution by teflon homogenizer. The homogenates of both tissues (kidney and liver) were centrifuged for 10 min at 3000*g*. The supernatant was collected and used for the estimation of oxidative stress parameters viz. reduced glutathione (GSH) level and thiobarbituric acid reactive substances (TBARS) level.

### Determination of serum lipid profile

Lipid profile consists of total cholesterol (TC), triglycerides (TG), high density lipoprotein (HDL), and low-density lipoprotein (LDL). Assessment of TC, TG, and HDL was done by commercially available kits using chemistry analyser (BeneSphera, USA). The level of LDL in serum sample was determined by using traditional Friedewald formula as described by Warnick et al. (1990). In this method, the calculated values of TC, HDL, and TG were used to determine the value of LDL.

$${\text{LDL}}\, = \,{\text{TC}} - {\text{HDL}} - ({\text{TG}}/{\text{5}}).$$

### Renal and liver function tests

Estimation of renal (creatinine, BUN, FeNa, and potassium) and liver (SGPT, SGOT, and ALP) function parameters in serum sample was also performed by commercially available kits using clinical chemistry analyser.

## Determination of oxidative stress parameters in kidney and liver

### Determination of GSH

The level of GSH in tissue was estimated by the method described by Beutler et al. (1963). Trichloroacetic acid (10% w/v) was mixed with tissue homogenate supernatant in 1:1 ratio. Reaction mixture was centrifuged at 12,000*g* for 10 min at 4 ℃. Resultant supernatant (0.5 ml) was mixed with 2 ml disodium hydrogen phosphate (0.3 M). Thereafter, 0.25 ml (0.001 M) of 5,5-dithio-bis-(2-nitro benzoic acid) (DTNB) (Loba Chemie, India) was added and absorbance was measured by spectrophotometer at 412 nm. DTNB was freshly prepared in 1% citric acid. Reduced glutathione (10–100 µM) was used as reference standard. Results were expressed as micromoles of reduced glutathione per mg of protein.

### Determination of TBARS

Lipid peroxidation in tissue results in TBARS. Quantitative measurement of TBARS was done as described by Niehaus and Samuelsson (1968). Under acidic conditions, malondialdehyde and other TBARS react with thiobarbituric acid to form a pink coloured chromophore, measured spectrophotometrically at 535 nm. In this method freshly prepared trichloroacetic acid (15%), hydrochloric acid (0.25 N), and thiobarbituric acid (0.375%) in the ratio of 1:1:1 were prepared. 1 ml of tissue homogenate was mixed thoroughly with 2 ml of trichloroacetic acid-hydrochloric acid-thiobarbituric acid reagent. The reaction mixture was kept in water bath at 100 ℃ for 15 min and centrifuged at 10,000*g* for 10 min. Colour in the reaction tubes was measured at 535 nm against reagent blank. 1,1,3,3- tetra methoxy propane (1–10 nM) was used as reference standard.

### Determination of SAG

The method described by Wang et al. (1998) was used to determine the SAG in tissue. Phosphate buffered saline (5 ml) containing 100 µM of nitro blue tetrazolium chloride (NBT) (Loba Chemie, India) was inoculated with 25 mg tissue and incubated at 37 ℃ for 90 min. After incubation reduction of NBT in the reaction was stopped by adding five ml HCl (0.5 M) followed by homogenization in 1 ml cocktail of 0.1 M sodium hydroxide, 0.1% sodium lauryl sulphate, and 40 mg/l diethylenetriaminepentaacetic acid. Centrifugation of the resulted mixture was done at 20,000*g* for 20 min. Pellet formed after centrifugation was dissolved in 1.5 ml pyridine and incubated at 80 ℃ for 90 min to extract formazan which formed after reaction of NBT with superoxide anions. Centrifugation was performed at 10,000*g* for 10 min. The absorbance of formazan was measured at 540 nm. Calculation of reduced NBT was performed by using following formula:

$$\text{amount\, of\,reduced\,NBT }= \frac{\text{A}\times \text{V}}{\text{T}\times \text{W}\times\upvarepsilon \times l}$$

where, A is absorbance, V is volume of pyridine, T is time for which the tissue was incubated with NBT, W is the weight of tissue used, ε is extinction coefficient and *l* is length of light path.

### Statistical analysis

All results were expressed as mean ± standard deviation. Statistical analysis was performed by using Graphpad prism software version 7.0. The significant difference within the groups was calculated by using one-way ANOVA, followed by Tukey’s multiple comparisons test. Results with P < 0.05 were considered as statistically significant. IC50 values were calculated by using probit analysis.

## Results

### Isolation and identification of endophytic fungi

**Fig.1:**
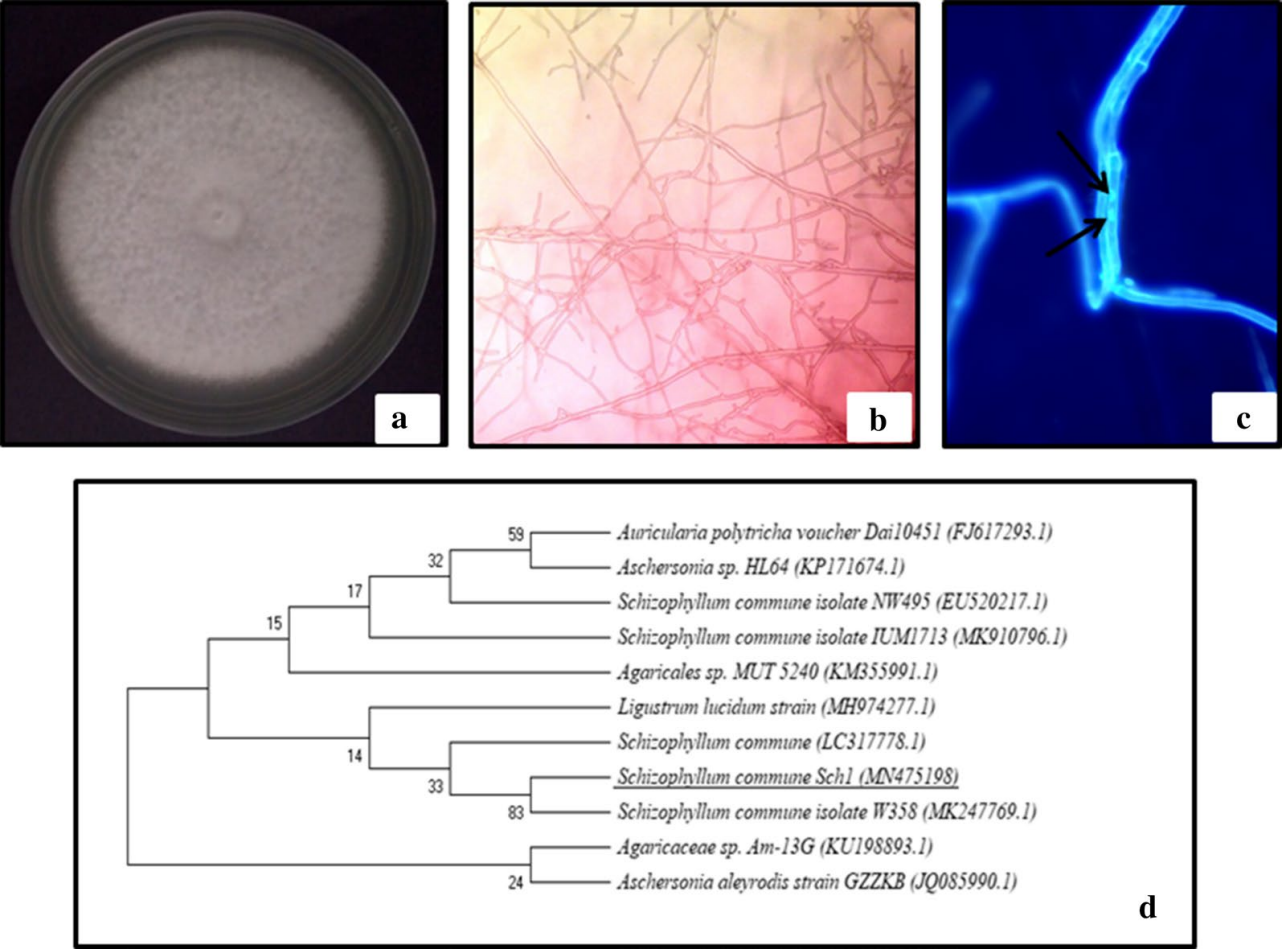
*S. commune* (Sch1) **a** colony morphology on PDA. **b** microscopic view (40X) **c** hyphae stained with DAPI under fluorescent microscope (100X). **d** ITS1-5.8S rDNA-ITS2 gene sequence based phylogenetic tree showing the taxonomic position of *S. commune* (Sch1). Phylogenetic analysis consisted of 11 nucleotide sequences. The evolutionary history was inferred using the Neighbor-Joining method. The optimal tree with the sum of branch length 1.58935111 is shown. All ambiguous positions were removed for each sequence pair. There were a total of 649 positions in the final dataset. Evolutionary analyses were conducted in MEGA X

In this study, an attempt was made to isolate endophytic basidiomycetes on medium supplemented with benomyl. Seventy three fungal cultures were isolated from different asymptomatic healthy plants and screened for their AGI activity. Forty fungal isolates were found to exhibit negligible (less than 20%) AGI activity, whereas 29 isolates showed inhibitory activity in the range of 40–60%. Good inhibitory activity above 80% was demonstrated by four endophytic cultures T1b, B2b, Sch1, and M1a isolated from *Ocimum tenuiflorum*, *Aegle marmelos, Aloe vera, Lawsonia inermis* respectively. The IC_50_ values of these cultures were also determined (Table 1). Morphological analysis revealed T1b, B2b, and M1a as species of *Alternaria.* Sch1 a non-sporulating mycelial culture was identified to be *Schizophyllum commune* on molecular basis by amplification of the ITS1-5.8S-ITS2 rDNA region. The isolate Sch1 was selected for further study. It exhibited more than 90% AGI activity with IC_50_ value of 425 µg/ml. The culture produced white coloured colonies on PDA plates reaching a diameter of 7.2 cm after six days of incubation at 30 ℃ (Fig. 1a). Hyphae were septate and profusely branched (Fig. 1b). After staining with DAPI, the hyphae were found to be dikaryotic as two nuclei in a single compartment were observed under fluorescent microscope (Fig. 1c). A sequence of 564 bps was obtained which on alignment with homologous nucleotide sequences revealed 100% similarity with *Schizophyllum commune*. The amplified ITS1-5.8S rDNA-ITS2 sequence has been submitted to GenBank under the accession number MN475198. On account of the observed morphological and molecular characteristics, strain Sch1 could be identified to be *Schizophyllum commune* (Fig. 1d). The isolate *Schizophyllum commune* Fr. (Sch1) has been deposited vide accession number NFCCI 4838 in National Fungal Culture Collection of India (NFCCI), Agharkar Research Institute, Pune, India.

**Table 1:** IC_50_ values of endophytic isolates possessing high (> 80%) AGI activity

Endophytic isolate	Host plant	IC_50_ (µg/ml)
T1b	*Ocimum tenuiflorum*	630
B2b	*Aegle marmelos*	763
Sch1	*Aloe vera*	425
M1a	*Lawsonia inermis*	582
Acarbose	–	76.6

### Effect of *S. commune* extract in STZ induced diabetic rats

As the isolate *S. commune* (Sch1) exhibited in-vitro antidiabetic effect, in-vivo antidiabetic studies were conducted in STZ induced diabetic rats.

### Hypoglycemic effect of *S. commune* extract

**Fig.2:**
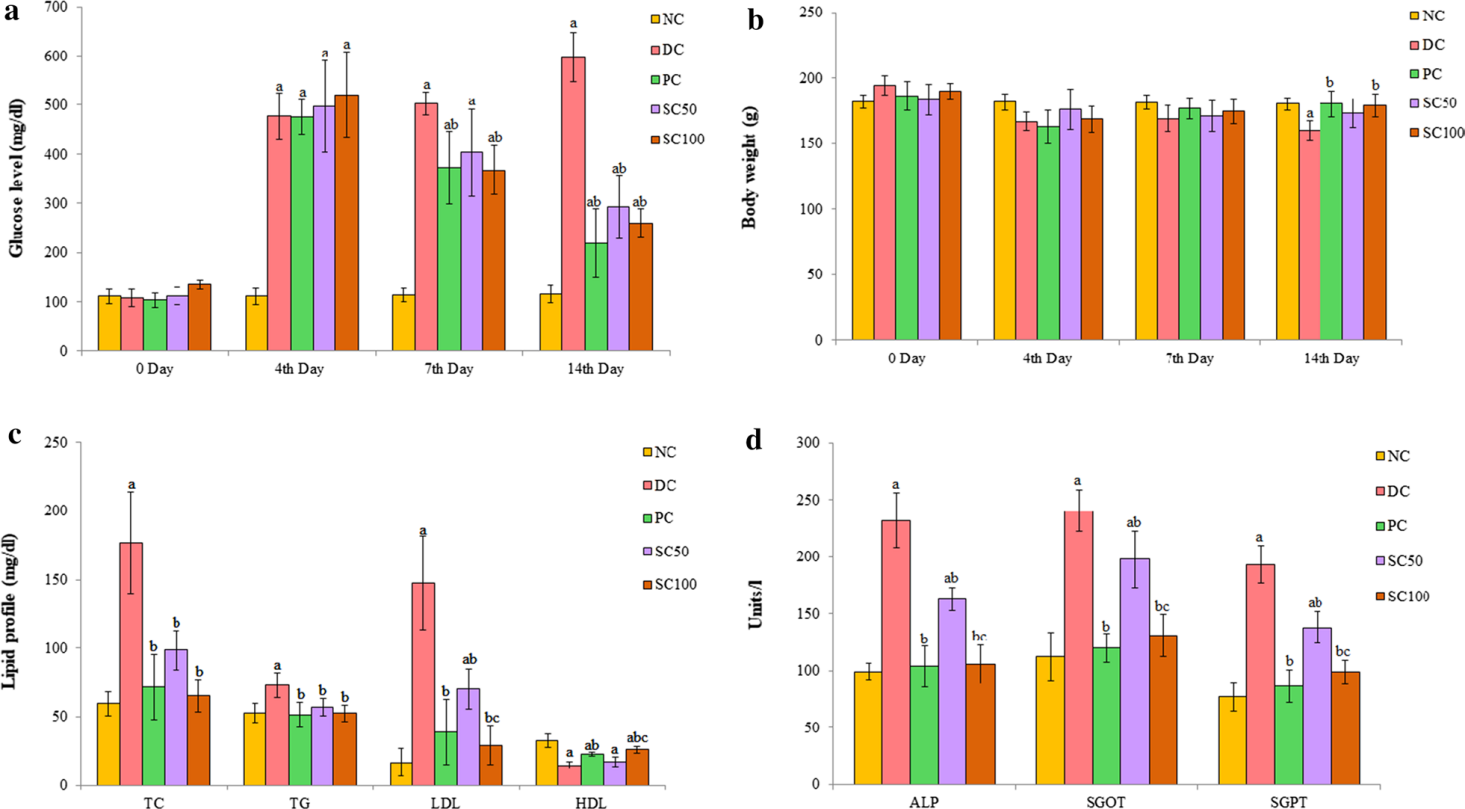
Effect of *S. commune* extract on **a** blood glucose level; **b** body weight; **c** lipid profile; **d** liver biochemical parameters in STZ induced diabetic rats. Data are expressed as mean ± S.D. (n = 5). NC: normal healthy control; DC: diabetic control; PC: positive control (STZ induced diabetic rats treated with reference drug acarbose (60 mg/kg body weight)); SC50 and SC100: STZ induced diabetic rats treated with *S. commune* extract (50 and 100 mg/kg body weight respectively). ^a^P < 0.05 Different control groups when compared with normal group. ^b^P < 0.05 Diabetic control group when compared with other treated groups. ^c^P < 0.05 *S. commune* extract treated groups when compared with each other

After the induction of diabetes in rats by intraperitoneal injection of STZ, the blood glucose level in experimental animals was significantly (P < 0.05) increased from the basal level as compared to the normal healthy control group. The level of blood glucose was found to be 597 ± 49.80 mg/dl in the diabetic control group at the end of the experimental period. A gradual decrease in the blood glucose level over time was recorded in diabetic rats treated with *S. commune* extract. At the end of the experiment, blood glucose level decreased significantly (P < 0.05) by 51% (292.2 ± 63.93 mg/dl) and 56% (259.78 ± 28.38 mg/dl) after treatment with 50 mg/kg and 100 mg/kg *S. commune* extract, respectively, as compared to the untreated diabetic control group (Fig. 2a). The effect of *S. commune* extract was comparable to standard drug acarbose, where 63% decrease in blood glucose level was recorded after 14 days experimental period.

### Effect of *S. commune* extract on body weight of diabetic rats

All STZ induced untreated diabetic rats manifested a significant (P < 0.05) loss of body weight when compared with the normal control group at the end of the experiment. The higher dose of *S. commune* extract was recorded to be more effective as significant (P < 0.05) gain of body weight after 14 days of treatment was documented when compared to the diabetic control group (Fig. 2b).

### Effect of *S. commune* extract on serum lipid profile

In diabetic rats, the levels of TC, LDL, and TG increased (P < 0.05), whereas HDL level decreased (P < 0.05) after 14 days as compared to normal healthy control. Treatment with *S. commune* extracts significantly (P < 0.05) improved the lipid profile in STZ induced diabetic rats (Fig. 2c). The effect was more pronounced at higher concentration (SC100) where the level of TC, TG, and LDL were decreased significantly (P < 0.05) by 63%, 28%, 80% respectively, whereas a significant increase of 78% in HDL level was observed as compared to the diabetic control group.

### Effect of *S. commune* extract on renal parameters

The present study considered the level of creatinine, BUN, potassium, and FeNa to measure the performance of the kidney. In STZ-induced diabetic rats, the level of these parameters was increased (P < 0.05) when compared with the normal control group. Although treatment with both SC50 and SC100 significantly (P < 0.05) reduced the level of creatinine, BUN, potassium, and FeNa as compared to the diabetic control group, the results were more pronounced at higher concentration of the extract. As evident in Table 2, the reduction obtained was also comparable with the positive and normal control group.

**Table 2:** Effect of *S. commune* extract on creatinine, BUN, FeNa, and potassium level in STZ induced diabetic rats

Groups	Creatinine (mg/dl)	BUN (mg/dl)	FeNa (%)	Potassium (mmol)
NC	0.756 ± 0.107	39.52 ± 9.123	3.214 ± 0.545	4.856 ± 1.374
DC	1.77 ± 0.165^a^	91.71 ± 12.781^a^	12.716 ± 2.847^a^	11.374 ± 2.304^a^
PC	0.852 ± 0.172^b^	50.656 ± 6.577^b^	5.513 ± 1.016^b^	4.264 ± 1.299^b^
SC50	1.188 ± 0.133^a,b^	69.666 ± 4.302^a,b^	7.588 ± 0.776^a,b^	8.054 ± 1.006^a,b^
SC100	0.872 ± 0.092^b,c^	51.444 ± 2.374^b,c^	5.226 ± 1.246^b^	4.528 ± 0.482^b,c^

Data are expressed as mean ± S.D. (n = 5). NC: normal healthy control; DC: diabetic control; PC: positive control (STZ induced diabetic rats treated with reference drug acarbose (60 mg/kg body weight)); SC50 and SC100: STZ induced diabetic rats treated with *S. commune* extract (50 and 100 mg/kg body weight respectively)

^a^P < 0.05 Different control groups when compared with normal group

^b^P < 0.05 Diabetic control group when compared with other treated groups

^c^P < 0.05 *S. commune* extract treated groups when compared with each other

### Effect of *S. commune* extract on oxidative stress parameters

Table 3 clearly displays the effect of *S. commune* extract on oxidative stress parameters. In this study, a significantly decreased level of GSH along with significantly increased levels of TBARS and SAG in kidney and liver of diabetic control were recorded. The imbalanced level of these oxidative stress markers was improved after treatment with *S. commune* extract. Treatment with higher concentration of *S. commune* extract (SC100) enhanced the GSH level in kidney and liver to 9.556 and 8.104 µmol/mg protein respectively, which in diabetic rats was observed to be 4.720 and 4.018 µmol/mg protein respectively. The levels of TBARS and SAG in the liver and kidney were also significantly (P < 0.05) reduced in diabetic rats treated with SC50 and SC100 as compared to untreated diabetic control.

**Table 3:** Effect of *S. commune* extract on oxidative stress parameters in STZ induced diabetic rats

Groups	GSH Kidney (µmol/mg protein)	GSH Liver (µmol/mg protein)	TBARS Kidney (nmol/mg protein)	TBARS Liver (nmol/mg protein)	SAG Kidney (pmol/min/mg tissue)	SAG Liver (pmol/min/mg tissue)
NC	11.01 ± 1.620	9.24 ± 1.088	0.398 ± 0.103	0.624 ± 0.128	5.04 ± 0.823	4.78 ± 1.304
DC	4.720 ± 0.486^a^	4.018 ± 0.623^a^	1.766 ± 0.296^a^	1.628 ± 0.378^a^	18.612 ± 1.868^a^	17.512 ± 1.571^a^
PC	11.892 ± 1.880^b^	8.168 ± 1.864^b^	0.532 ± 0.127^b^	0.602 ± 0.057^b^	6.19 ± 0.862^b^	7.228 ± 0.640^a,b^
SC50	6.862 ± 0.768^a^	5.092 ± 0.690^a^	1.118 ± 0.103^a,b^	1.038 ± 0.082^a,b^	13.104 ± 1.159^a,b^	12.832 ± 1.007^a,b^
SC100	9.556 ± 1.032^b,c^	8.104 ± 1.647^b,c^	0.604 ± 0.105^b,c^	0.732 ± 0.084^b^	10.21 ± 1.239^a,b,c^	10.216 ± 0.746^a,bc^

Data are expressed as mean ± S.D. (n = 5). NC: normal healthy control; DC: diabetic control; PC: positive control (STZ induced diabetic rats treated with reference drug acarbose (60 mg/kg body weight)); SC50 and SC100: STZ induced diabetic rats treated with *S. commune* extract (50 and 100 mg/kg body weight respectively)

^a^P < 0.05 Different control groups when compared with normal group

^b^P < 0.05 Diabetic control group when compared with other treated groups

^c^P < 0.05 *S. commune* extract treated groups when compared with each other

### Effect of *S. commune* extract on liver function parameters

In the diabetic control group, the level of ALP, SGOT, and SGPT increased (P < 0.05) as compared to the normal healthy control. Diabetic rats treated with *S. commune* extract evinced significant (P < 0.05) reduction in the level of SGOT, SGPT, and ALP after 14 days of treatment.

As evident from the results (Fig. 2, Tables 2, 3) both the concentrations showed improvement in the observed biochemical parameters. Dose dependent effects were also observed for the majority of the parameters viz*.* LDL, HDL, ALP, SGOT, SGPT, creatinine, BUN, potassium, GSH, SAG, TBARS (kidney). In case of TG, TC, FeNa, TBARS (liver) comparable improvement was obtained with both the doses.

An attempt to determine the nature of the compounds present in the extract was also made by using phytochemical analysis. *S. commune* extract was found to contain phenolic and terpenoid compounds. Presence of the phenols was indicated by the appearance of brown-reddish violet colour in fast blue B test and confirmed by the formation of violet colour in ferric chloride test, while for terpenoids an intense reddish-brown colour was developed at the interface when sulphuric acid was added in chloroform dissolved fungal extract. Lack of development of orange brown colour on spraying with dragendorff reagent indicated the absence of alkaloids. The absence of amines was also determined as no pink or purple colour spots were obtained when sprayed with ninhydrin.

## Discussion

The present study aimed at the isolation of endophytic basidiomycetes and evaluation of their antidiabetic potential. A survey of literature revealed that, despite their potential as a natural source of bioactive metabolites, endophytic basidiomycetes have not been extensively isolated and screened for their bioactivities. Endophytic basidiomycetes account only for 10% of the total endophytic population isolated from different plants (Rana et al. 2019). Isolation of endophytic basidiomycetes was carried out on medium supplemented with benomyl. Benomyl is a fungicide which interferes in the process of mitosis in fungi by targeting microtubules. It can act as a selective agent for the isolation of basidiomycetes as some strains possess a mutation in the *β*1- tubulin gene which makes them resistant to benomyl (Matsuo et al. 1999). Even though the medium used in isolation is recommended for the basidiomycetes, members of the order pleosporales, to which *Alternaria* belongs, are reported to show resistance against benomyl (Summerbell 1993). This could account for a high number of endophytic *Alternaria* sp. obtained in the present study*.* Preliminary screening for determining antidiabetic potential was carried out by the ability of the isolates to produce AGI. On the basis of in-vitro AGI activity, a fungal culture Sch1 identified to be *Schizophyllum commune* was selected. *S. commune*, a basidiomycete commonly known as split gill fungus, belongs to the family schizophyllaceae of the class agaricomycetes. It is an edible mushroom consumed in different parts of the world mainly in Mexico, Peninsular Malaysia, Nagaland (India), and the Republic of the Congo (Guzmán 2008; Lee et al. 2009; Tanti et al. 2011; Kamalebo et al. 2018). It has also been reported as an endophyte from various plants viz*. Tectona grandis, Musa* spp., and *Cannabis sativa* by various researchers (Chareprasert et al. 2006; Assunçao et al*.* 2010; Qadri et al. 2013). A survey of literature has revealed the traditional use of *S. commune* as therapeutic for headache, indigestion, inflammation, intestinal pain, obesity, rheumatism, and weakness (Guzmán 2008; Kamalebo et al. 2018). In addition, *S. commune* also exhibits antioxidant, antiviral, antitumor, and immunomodulating activities (Ooi and Liu 2000; Klaus et al. 2011). Although α-glucosidase inhibitors from different endophytic fungi have been reported (Ramdanis et al. 2012; Singh and Kaur 2016; Kaur et al. 2019) to the best of our knowledge this is the first study revealing the AGI activity of endophytic *S. commune*.

As the culture exhibited in-vitro antidiabetic activity, its in-vivo effect was also studied in STZ induced diabetic rats. STZ is the most commonly used drug to induce diabetes in experimental animals. GLUT-2 transporter in the plasma membrane transports STZ to the pancreatic beta cells. Accumulation of STZ in beta cells results in cell necrosis by DNA alkylating activity which in turn reduces the synthesis and release of insulin, responsible for hyperglycemia (Lenzen 2008). Oral administration of *S. commune* extract displayed significant improvement in glucose homeostasis of diabetic rats. The lowering in blood glucose level could be due to the AGI potential of *S. commune* extract. α-Glucosidase is a carbohydrate hydrolysing enzyme present in the brush border of the intestine. Carbohydrate hydrolysing activity of this enzyme is mainly responsible for postprandial hyperglycemia. α-Glucosidase inhibitors such as acarbose and voglibose help to reduce the blood glucose level by expanding the carbohydrate absorption duration and have been used since a long time for the treatment of diabetes mellitus (Van De Laar et al. 2005).

Another clinical feature of diabetes mellitus is body weight loss, due to tissue wasting caused by poor glycemic control which results in increased protein and fat metabolism in diabetic rats (Adeoye et al. 2017). A significant increase in the body weight of diabetic rats treated with fungal extract was recorded at the end of the experimental period. Diabetes mellitus is also characterized by dyslipidemia which accounts for elevated levels of TC, TG, LDL, and lower level of HDL (Bhowmik et al. 2018.) In diabetic patients, dyslipidemia is a major cause of cardiovascular disease. There are several factors that are responsible for disturbed lipid profile in diabetic patients viz. effect of insulin on the production of apoprotein in the liver, lipoprotein lipase regulation, activity of cholesteryl ester transfer protein, and action of insulin on muscle and adipose (Goldberg 2001). Treatment with *S. commune* extract showed significant improvement in lipid profile. Diabetic nephropathy is another major complication of diabetes mellitus. Among diabetic patients, approximately 40% require dialysis on a regular basis. Diabetes associated glomerular damage results in chronic renal insufficiency (Patschan and Müller 2016). Creatinine, BUN, potassium, and FeNa have been used as markers of kidney performance (Gowda et al. 2010). A significant increase in these parameters was documented in untreated diabetic rats compared with normal control. This could be the result of kidney damage caused by diabetes. Diabetic rats treated with *S. commune* extract produced significant improvement in kidney parameters level after 14 days experimental period.

It has been recognized that oxidative stress plays a crucial role in the pathogenesis of diabetes related vasculopathy, retinopathy, neuropathy, and nephropathy along with gastrointestinal complications. The natural antioxidant defence system is also depressed when acute elevation in glucose level occurs (Giugliano et al. 1996; Kashyap and Farrugia 2011). GSH (non-protein thiol) is a potent antioxidant existing nearly in all living cells and is used as a redox imbalance biomarker at cellular level. Disturbed GSH profile is embroiled in β-cell dysfunction along with pathogenesis of long-term diabetes assisted complications (Tiwari et al. 2013). Free radical activity has been reported to increase in diabetes mellitus. These free radicals cause lipid peroxidation which plays a vital role in atherosclerosis, aging, and late complication of diabetes mellitus. TBARS determination was performed to measure the level of lipid peroxidases (Griesmacher et al. 1995). Hyperglycemia also results in superoxide anion generation, which ultimately causes oxidation of proteins, nitration of amino acids, and initiates lipid peroxidation (Wright et al. 2006). In the present study, an imbalance in oxidative stress parameters documented in untreated diabetic control was restored significantly after treatment of the diabetic rats with *S. commune* extract.

Transaminases like SGPT, SGOT play an important role in the breakdown of amino acids into α-keto acid. These enzymes are specific indicators of liver function. In hepatocyte injury, the level of these enzymes increases which enhances the rate of gluconeogenesis and ketogenesis (Onaolapo and Onaolapo 2012). Another enzyme ALP can also be used to monitor liver functioning. Diabetes is responsible for a large number of liver disorders viz. elevated liver enzymes, hepatocellular carcinoma, fatty liver disease, cirrhosis, and acute liver failure (Tolman et al. 2007). In this study diabetic rats treated with *S. commune* extract revealed a significant reduction in the level of SGOT, SGPT, and ALP. It is evident from the results obtained that the administration of *S. commune* extract significantly exerted a beneficial effect on all the observed biochemical parameters.

Phytochemical analysis to detect the nature of the compounds revealed the presence of phenolic and terpenoids which could be responsible for the observed effect. Phenolic and terpenoid compounds have been reported to manifest antihyperglycemic and antioxidant effects in diabetic rats (Gandhi et al. 2011; Germoush et al. 2020). Reports of antidiabetic potential of various basidiomycetes and compounds produced by them are available in literature. Phenolic compounds with AGI activity have been reported in mushrooms (Liu et al. 2012). Potent α-glucosdiase inhibitory terpenoids were characterized from submerged grown culture of *Inonotus obliquus* (Ying et al. 2014). Different species of *Ganoderma* are reported to produce various types of terpenoids and polysaccharides with the ability to cure diabetes and insulin resistance (Wińska et al. 2019). β-Glucans and oligosaccharides isolated from *Agaricus blazei* were found to exert hypoglycemic effect in diabetic rats (Kim et al. 2005). Antidiabetic β-glucans and polysaccharide-peptide have been isolated from species of *Pleurotus* (Kanagasabapathy et al. 2012; Chen et al. 2015). Although various basidiomycetes have been reported to exhibit antidiabetic activity (Jeong et al. 2010; Ng et al. 2015), this is the first report highlighting the therapeutic potential of *S. commune* in the management of diabetes.

It can be concluded that *S. commune* can be exploited as a source of compounds that can have potential in the management of diabetes. The study also reveals the importance of endophytic basidiomycetes as sources of bioactive metabolites.

## Data Availability

All data generated or analysed during this study are included in this published article.

## Abbreviations

STZ: Streptozotocin
PDA: Potato dextrose agar
AGI: α-Glucosidase inhibitory
p-NPG: *p*-Nitrophenyl-alpha-d-glucopyranoside
BUN: Blood urea nitrogen
FeNa: Fractional excretion of sodium
SGPT: Serum glutamic pyruvic transaminase
SGOT: Serum glutamic-oxaloacetic transaminase
ALP: Alkaline phosphatase
GSH: Reduced glutathione
TBARS: Thiobarbituric acid reactive substances
TC: Total cholesterol
TG: Triglyceride
HDL: High density lipoprotein
LDL: Low-density lipoprotein
DTNB: 5,5-Dithio-bis-(2-nitro benzoic acid)
NBT: Nitro blue tetrazolium chloride
